# A cross-sectional examination of the simultaneous association of four emotion regulation strategies with abnormal eating behaviours among women in Japan

**DOI:** 10.1186/s40337-021-00477-7

**Published:** 2021-09-28

**Authors:** Yasuo Murayama, Aiko Ohya

**Affiliations:** 1 Institute of Human and Social Sciences, Kanazawa University, Kakuma-machi, Kanazawa, Ishikawa 921-1192 Japan; 2 Faculty of Psychology, Doshisha University, 1-3 Tatara, Miyakodani, Kyotanabe-shi, Kyoto 610-0394 Japan

**Keywords:** Emotion regulation strategies, Rumination, Abnormal eating behaviours/attitudes, Psychological distress, Cultural differences, Women

## Abstract

**Background:**

Research has suggested an association between emotion regulation strategies (ERSs) and abnormal eating behaviours/attitudes (AEB), and many studies have examined the association of one particular ERS with AEB. Additionally, different ERSs are reported to be strongly correlated with each other. Therefore, the associations between an individual ERS and AEB, reported previously, may be spurious. The present cross-sectional study aims to examine the simultaneous associations of four ERSs (brooding, reflection, expressive suppression, cognitive reappraisal) with AEB in a sample of women in Japan.

**Methods:**

The participants comprised 1528 Japanese women (*M*_age_ = 40.65 years, *SD*_age_ = 10.22 years, range 21–59). They self-reported the frequency at which they use these ERSs, their levels of AEB (i.e. drive for thinness, bulimic symptoms), and the confounding variables (e.g. psychological distress and BMI) online. AEB was measured using the Japanese version of the 91-item Eating Disorder Inventory; brooding and reflection were measured using the Japanese version of the Rumination Response Scale; individual differences in the use of reappraisal and expression suppression was measured using the Japanese version of the Emotion Regulation Questionnaire (J-ERQ); and participants’ psychological distress was assessed using the Kessler 6 Japanese version (K6-J).

**Results:**

Correlation analyses revealed that all ERSs were positively correlated with AEB. However, regression analyses revealed inconsistent findings. In the regression model, after controlling for the confounding variables, only brooding indicated a positive association with the drive for thinness. Regarding bulimic symptoms, all ERSs showed a positive association, except reappraisal, which had a weak, negative association.

**Conclusion:**

These results suggest that brooding is related to the symptom levels of both eating disorders among women, whereas, the other ERSs are related to those of bulimic symptoms only. However, further research is required to clarify the causal relations between AEB and ERSs.

## Plain English Summary

This study aimed to cross-sectionally examine the association between four emotion regulation strategies and major symptoms of eating disorders. Participants comprised 1528 Japanese women in their 20s to 50s. They self-reported the frequency of using the strategies, levels of symptoms of eating disorders, and other information online. The results showed that the symptoms relating to overeating were more likely to be associated with the four strategies than the symptoms relating to food restriction. Further, one of the strategies—a repetitive negative thinking pattern—was more likely to be associated with the two symptoms of eating disorders: drive for thinness and bulimic symptoms. These results suggest that improving one’s thinking style may be associated with decreased levels of these symptoms. Therefore, providing training in the community to enhance mindfulness—reportedly associated with decreasing the thinking style–can help prevent AEB among adult women. Considering these findings, yoga exercises, which involve meditation-like mindfulness training, can be regarded as effective measures against psychological distress.

## Background

Abnormal eating behaviours/attitudes (AEB), which are symptomatic of eating disorders, are likely to develop among adolescents, especially girls [1]. However, available data have indicated that some adult women have high levels of AEB. One study on women aged 28 to 39 reported a prevalence rate of 1.9% for anorexia nervosa and 2.9% for bulimia nervosa [2]. Another study demonstrated that 8% of women aged around 40 years frequently engaged in binge eating [3].

Research in non-Western countries has also demonstrated the prevalence of AEB among women. One cross-sectional study in Japan [4] reported that 1.5% of working women in their 20s and 30s showed above cut-off scores on the Eating Attitudes Test-26 (EAT-26). Another community-based study using the same scale revealed that 2.4% of Japanese women aged 20 to 39 demonstrated severe AEB [5]. These findings suggest that AEBs are prevalent among adult women in modern society.

Recent empirical evidence suggests that negative affect often precedes the occurrence of AEB. Several cross-sectional and prospective studies have found that elevated negative affect was associated with a greater likelihood of AEB [6, 7]. Another prospective study has revealed that depression predicts elevated levels of AEB [8]. Similarly, another study suggests that depressive and anxiety disorders often precede the development of eating disorders [9]. These findings indicate that variables that regulate negative affect, adaptively or maladaptively, may be potential factors contributing to AEB.

Emotion regulation strategies (ERSs); that is, strategies to alleviate, maintain, or elevate positive or negative emotions [10] can be used to modulate negative affect. A growing number of studies have identified several major ERSs (i.e. cognitive reappraisal, expressive suppression, and rumination) that can contribute to levels of psychopathology. Adaptive ERSs likely attenuate levels of psychopathology, whereas, maladaptive ERSs, which often occur automatically (i.e. automatic maladaptive ERSs), develop and/or maintain the levels [11]. Previous research has reported that automatic maladaptive ERSs are transdiagnostic risk factors [12]. In fact, a study among adolescent girls reported that an automatic maladaptive ERS predicted the onset of eating disorders [13]. Thus, examining the relationships between ERSs and AEB may contribute to the development of more effective prevention and treatment procedures for eating disorders among adult women. However, a limited number of studies have examined the associations between these ERSs and AEB in adult women. Thus, this study sought to examine the associations of adaptive/maladaptive ERSs with AEB among women.

Cognitive reappraisal, or generating positive interpretations of a stressful situation to reduce distress [14], is a major adaptive ERS. Previous research has revealed that greater use of reappraisal is related to elevated positive affect, decreased negative affect, and less severe depression [15]. Regarding the association between reappraisal and AEB, prior research has indicated mixed findings. An experimental study in individuals with eating disorders demonstrated that reappraisal reduced the likelihood of engaging in AEB [16]. One self-report study indicated that middle-aged women with more frequent use of reappraisal demonstrated decreased levels of food restraint, whereas levels of reappraisal were not significantly associated with bulimic symptoms [17]. Another cross-sectional study showed that the correlation directions between reappraisal and AEB differed based on the type of eating disorders, such that individuals with anorexia nervosa indicated negative correlation, whereas, those with bulimia nervosa indicated a positive one [18].

One of the major automatic maladaptive ERSs, which is incompatible with reappraisal, is expressive suppression, or the effort to inhibit ongoing emotion-expressive behaviour [14]. Previous research in Western cultures has reported that frequent use of expressive suppression may lead to paradoxical increases in negative affect and depression [19]. On the contrary, one study among individuals in Hong Kong demonstrated no significant link between expressive suppression and depressive symptoms [20]. Regarding AEB, multiple cross-sectional studies in the West have shown that expressive suppression is positively associated with AEB in patients with eating disorders— both adolescents and adults [18, 21]. However, one experimental study in patients with eating disorders showed that the suppression of negative emotion failed to induce overeating [22]. Meanwhile, there is little available data regarding associations between expressive suppression and AEB among individuals in Eastern cultures.

Another well-researched automatic maladaptive ERS is rumination. There are two types of rumination: brooding and reflection. Brooding involves repetitive negative thinking patterns regarding reasons for negative mood, whereas reflection involves an active inward focus on distress to solve the negative situation [23]. Several cross-sectional studies have demonstrated that brooding, but not reflection, was positively associated with AEB [24, 25]. However, one cross-sectional investigation showed mixed findings: brooding was positively associated with AEB in healthy individuals, whereas reflection was positively associated with it in patients with eating disorders [24].

In summary, prior studies have yielded inconsistent findings regarding the associations between ERSs and AEB. Furthermore, although several empirical studies have indicated that individuals typically use multiple ERSs simultaneously [26], many studies have examined the associations of a single ERS with AEB. Additionally, based on empirical evidence that some ERSs are strongly correlated with each other [27], any significant association between an individual ERS and AEB reported in prior studies might stem from spurious correlations via the effects of other ERSs. Furthermore, several studies have demonstrated that aspects of individuals’ cultural background, such as a tendency toward individualism or collectivism in social organisation, affect the functions of ERSs [28]. Therefore, associations of ERSs with AEB may differ across cultures. Despite this, only a few studies have examined the associations between ERSs and AEB in the East, including Japan. Finally, although there are several components to AEB, such as the drive for thinness or bulimic symptoms, most previous research has examined the associations of a single ERS with overall AEB. There are few available data demonstrating associations between ERSs and the specific symptomatology of eating disorders.

Given these limitations, the associations between each ERS and AEB reported in previous research may be spurious. In addition, empirical findings reported in the West may not be applicable to individuals in the East. From these perspectives, the limitations of previous research may mislead researchers and practitioners in AEB-related research and treatments. Thus, the current cross-sectional study sought to examine the simultaneous associations of four ERSs (i.e. reappraisal, expressive suppression, brooding, and reflection) with specific aspects of AEB in a sample of women in Japan, controlling for body mass index (BMI) and psychological distress, such as depression or anxiety, which is associated with AEB [25]. Therefore, this study may provide implications as to which ERSs should be targeted to prevent the development of AEB in adult women. Since previous research has shown that the onset of eating disorders seldom occurs in individuals over 60 years of age [29], we recruited women in their 20s to 50s.

Previous studies across cultures demonstrated similar findings in the associations of all ERSs, except expressive suppression, with mental health problems, such that elevated rumination and decreased reappraisal are associated with greater levels of depression [20]. A previous study revealed a positive association between expressive suppression and psychological distress in individuals in Western cultures, but a non-significant association in those in East Asian [20]. Aligning with this finding, a cross-sectional study in Japan reported non-significant correlations between expressive suppression and adaptive psychological functioning (i.e. self-esteem and subjective happiness) [30]. Moreover, regarding AEB, studies in Japan have revealed that cognitive reappraisal is negatively associated with AEB [31], and that individuals diagnosed with eating disorders indicated higher frequency in use of rumination than healthy controls [32]. Based on these findings, we expected that the ERSs, except expressive suppression, would be associated with AEB. As such, higher levels of rumination and lower levels of reappraisal would be associated with greater AEB, whereas the use of expressive suppression would not be associated with AEB.

## Methods

### Participants

The participants comprised women in Japan aged between 20 to 50 years, registered as members (i.e. possible respondents) at a research company. As of April 2019, 2.2 million individuals in Japan had registered as members of the research company. There were two criteria for participation in this study: being female and being within a specific age range (20–59 years old). Because it was not the aim of this study to examine the prevalence of eating disorders, diagnosis with an eating disorder was not among the participation criteria of this study. Based on national data regarding age and region distribution of the female population [33], 2000 women were randomly selected from among the company’s possible respondents, using a random number table of their registration numbers. For example, consistent with the national data reporting that approximately 9.5% of the total female population in Japan are women aged in their 40s living in the Kanto area, we recruited 190 women (9.5%) in their 40s living in the Kanto metropolitan region out of the total sample (*n* = 2000). Only those who agreed to participate were recruited for this study. As 472 participants refused to participate, 1528 women (*M*_age_ = 40.65 years, *SD*_age_ = 10.22 years, range 21–59) participated in the online survey. The data were collected in September 2019 and there was no difference in the rate of participants who did not agree to participate between the regions or the age categories (i.e. 20s, 30s, 40s, and 50s; *χ*^2^(21) = 17.98, *n.s.*). Table 1 illustrates the demographic data of the sample.

**Table 1:** Demographic characteristics of the sample

Variable	*n*	(%)
*Age*		
20s	317	(20.7)
30s	391	(25.6)
40s	458	(30.0)
50s	362	(23.7)
*Household income*		
< 1,000,000 yen	63	(4.1)
1,000,000–1,999,999 yen	107	(7.0)
2,000,000–2,999,999 yen	178	(11.6)
3,000,000–3,999,999 yen	216	(14.1)
4,000,000–4,999,999 yen	199	(13.0)
5,000,000–6,999,999 yen	310	(20.3)
7,000,000–9,999,999 yen	286	(18.7)
10,000,000–14,999,999 yen	121	(7.9)
15,000,000–19,999,999 yen	31	(2.0)
≥ 20,000,000 yen	17	(1.1)
*Partnered status*		
Single/never married	480	(31.4)
Married	916	(59.9)
Divorced	132	(8.6)
*Children*		
Yes	777	(50.9)
No	751	(49.1)
*Weight group (BMI)*		
Underweight (< 18.5)	291	(19.0)
Normal (18.5–24.9)	1063	(69.6)
Overweight (> 25.0)	174	(11.4)

BMI: Body Mass Index

### Measures

#### Japanese version of the 91-item Eating Disorder Inventory

AEB was measured using the Japanese version of the 91-item Eating Disorder Inventory (J-EDI-91) [34], which was developed based on the Eating Disorder Inventory-2 [35]. Previous studies have indicated that the J-EDI-91 has high reliability and validity [34]. In this study, the drive for thinness and bulimia subscales in J-EDI-91 were used to measure symptoms of anorexia and bulimia, respectively. These subscales consist of eight (e.g. “I think about dieting”) and seven (e.g. “I stuff myself with food”) items measured on a six-point scale (1 = *never* to 6 = *always*), with the most dysfunctional response being scored as 3, followed by 2, and then 1, and the other responses as 0. Higher scores indicate more severe symptoms. In the current study, Cronbach’s alphas were 0.922 and 0.887 for drive for thinness and bulimia subscales, respectively.

#### Japanese version of the Rumination Response Scale

Brooding and reflection were assessed using the Japanese version of the Rumination Response Scale (J-RRS) [36], developed from the original RRS [37]. This scale has two subscales, namely brooding (e.g. “I think ‘Why do I always react this way?’”) and reflection (e.g. “I analyse recent events to try to understand why I am depressed”), each containing five items rated on a four-point scale (1 = *almost never*, 4 = *almost always*). Higher scores indicate more frequent use of these ruminative strategies. The J-RRS has demonstrated high reliability and validity [36]. In the current study, Cronbach’s alphas were 0.924 and 0.887 for Brooding and Reflection, respectively.

#### Japanese version of the Emotion Regulation Questionnaire (J-ERQ)

The Japanese version of the Emotion Regulation Questionnaire (J-ERQ) [30] was used to measure individual differences in the use of reappraisal and expression suppression. The J-ERQ, developed from the original ERQ [15], contains ten items grouped into two subscales: Reappraisal (six items, such as “I control my emotions by changing the way I think about the situation I’m in”) and Suppression (four items, such as “I control my emotions by not expressing them”), rated on a seven-point scale (1 = *strongly disagree* to 7 = *strongly agree*). Higher scores indicate more frequent use of these strategies. The J-ERQ has been demonstrated to have high reliability and validity [30]. In the current study, Cronbach’s alphas were 0.939 and 0.880 for the reappraisal and suppression subscales, respectively.

#### Kessler 6 Japanese version

The Kessler 6 Japanese version (K6-J) was used to measure participants’ psychological distress, such as depression. The K6-J was developed from the original K6—a preliminary screening tool for a wide range of mental disorders to measure psychological distress in individuals [38]. The K6-J contains six items (e.g. “During the past 30 days, about how often did you feel nervous?”) rated on a five-point scale (1 = *all of the time* to 5 = *none of the time*). Higher scores indicate greater psychological distress. The K6-J has demonstrated high validity [39]. In the current study, Cronbach’s alpha was 0.924.

#### Body mass index

Participants’ height (m) and body weight (kg) were measured to calculate BMI (weight [kg]/height^2^ [m^2^]).

### Procedure

All the aforementioned items and questionnaires were answered on the web. Before answering the items, each participant consented to participate in the current study. All the participants’ identities have been anonymised. The Ethical Committee of Kobegakuin University approved this study (approval number: HP-18-09).

### Data analysis

First, as part of the descriptive statistics, zero-order collations between each measured variable were examined. However, the correlation analysis cannot exclude the possibility of spurious correlations because the analysis does not remove the effects of the other variables on the association between the two variables of interest. Therefore, to examine individual associations between each of the four ERSs and AEB while controlling the effects of the other ERSs, hierarchical multiple regression analyses were performed, which also explained the extent to which the ERSs accounted for the level of AEB. In the analyses, age, BMI, and psychological distress were entered in Step 1 and the ERSs in Step 2. All quantitative data were standardised. Further, to examine the association of each ERS with a specific eating pathology, we stratified the results by type of AEB (i.e. drive for thinness and bulimic symptoms). The significance level was set at *p* < 0.05. Predictive Analytics SoftWare (PASW) Statistics (SPSS ver. 24.0) was used for all analyses.

## Results

### Descriptive statistics

Means, standard deviations, and zero-ordered correlations among the variables of interest are shown in Table 2. The coefficient of skewness of each quantitative variable was under 3, indicating that the quantitative variables were normally distributed [40]. The score of the subscale drive for thinness (*M* = 4.52, *SD* = 6.24) was somewhat lower than the finding in a prior study among Japanese healthy adolescent girls and young women (Shimura et al., 2003; *M* = 5.76, *SD* = 0.25), while that of the subscale bulimia (*M* = 1.69, *SD* = 3.66) was nearly equal to prior study’s finding (Shimura et al., 2003; *M* = 1.79, *SD* = 0.14).

**Table 2:** Pearson’s correlations, means, and standard deviations of all variables

		1	2	3	4	5	6	7	8	*M*	*SD*	Skew
1	Age	–								40.65	10.22	− 0.04
2	BMI	.111***	–							21.18	3.35	2.05
3	K6 Psychological Distress	− .069**	.031	–						4.26	5.28	1.55
4	EDI Drive for Thinness	− .104***	.208***	.357***	–					4.52	6.24	1.33
5	EDI Bulimia	− .109***	.124***	.461***	.667***	–				1.69	3.66	2.97
6	RRS Brooding	− .088**	.049	.696***	.410***	.463***	–			8.33	4.08	1.22
7	RRS Reflection	− .141***	− .008	.625***	.335***	.433***	.814***	–		7.39	3.32	1.59
8	ERQ Reappraisal	.054*	.004	.088**	.101***	.053*	.170***	.209***	–	21.87	8.66	− 0.48
9	ERQ Suppression	.096***	.003	.171***	.124***	.140***	.208***	.226***	.697***	13.09	5.54	− 0.16

K6: Kessler 6; EDI: Eating Disorder Inventory; RRS: Rumination Response Scale; ERQ: Emotion Regulation Questionnaire

*p < .05, **p < .01, ***p < .001

Each ERS was positively correlated with drive for thinness, with small effect sizes for reappraisal and suppression and medium to large effect sizes for brooding and reflection. Furthermore, all ERSs were significantly and positively correlated with bulimic symptoms, with a very weak correlation for reappraisal, small for suppression, and medium to large for brooding and reflection. The control variables were significantly correlated with both AEBs; age showed a negative correlation, while BMI and psychological distress showed a positive correlation. Further, the four ERSs were positively correlated with each other.

### Simultaneous associations between the ERSs and AEB

Table 3 shows the results of the hierarchical multiple regression analyses. Variance inflation factors in the analyses were under 3.61; none of the data were affected by multicollinearity. Regarding drive for thinness, adding the four ERSs in the second step significantly accounted for 4.7% of the variance. Brooding indicated a significant positive association with a moderately sized coefficient. In Step 2, all the control variables showed significant associations. The size of the coefficient for psychological distress was medium, while those of age and BMI were small.

**Table 3:** Hierarchical regression analysis predicting abnormal eating attitudes/behaviours

Step	Variables	Drive for thinness				Bulimic symptoms			
		*B*	β	*R*^2^	Δ*R*^2^	*B*	β	*R*^2^	Δ*R*^2^
1				.177	.177***			.233	.233***
	Age	− 0.105	− 0.104***			− 0.094	− 0.091***		
	BMI	0.218	0.209***			0.128	0.121***		
	Psychological distress	0.348	0.343***			0.466	0.451***		
2				.224	.047***			.280	.047***
	Age	− 0.099	− 0.098***			− 0.080	− 0.078**		
	BMI	0.208	0.200***			0.125	0.118***		
	Psychological Distress	0.142	0.141***			0.252	0.244***		
	Brooding	0.299	0.301***			0.173	0.171***		
	Reflection	− 0.022	− 0.022			0.129	0.127***		
	Reappraisal	0.021	0.021			− 0.098	− 0.096**		
	Suppression	0.037	0.037			0.110	0.108***		

BMI: Body Mass Index

*p < .05, **p < .01, ***p < .001

Regarding bulimic symptoms, adding the ERSs in the second step significantly contributed to 4.7% of the variance: all the ERSs showed significant associations. Brooding, reflection, and expression suppression were positively associated with bulimic symptoms with small effect sizes. In contrast, reappraisal showed a unique negative association with weak effect size. All the control variables showed significant associations. The size of the coefficient for psychological distress was medium to large, and those of the other variables were small.

## Discussion

Considering the limitations of previous research (i.e. few studies examining the simultaneous association of multiple ERSs with AEB and fewer studies in the East), the current study simultaneously examined the associations between the four major ERSs and AEB among women in Japan. Although the correlational analyses indicated positive correlations between each of the four ERSs and AEB, the regression models showed some inconsistent results. In fact, regression analyses indicated that all ERSs were significantly associated with bulimic symptoms, whereas only brooding was associated with the drive for thinness.

### Associations between reappraisal and AEB

Correlation and multiple regression analyses showed that the coefficients between reappraisal and anorexic/bulimic symptomatology were very low, such that the standardised partial coefficients were under 0.1, although some of the associations were significant. These findings suggest that reappraisal contributes little to AEB levels among women in the East, which is inconsistent with empirical data found in the West [16]. The difference may be related to cross-cultural differences in the usage of reappraisal. In line with the definition of reappraisal [14], Westerners are likely to use reappraisal after experiencing negative events due to the Western cultural script to encourage individuals to maximise positive emotions and minimise negative ones [41]. However, the dominant script in Eastern culture is related to the notion that individuals prefer to seek and keep a middle way with a balance between positive and negative aspects, including emotion [42]. Therefore, individuals in the Eastern culture may use reappraisal when experiencing positive and negative events. Supporting this contention, a study by Deng, Sang, and Luan [43] indicated that by considering long-term success, Chinese adolescents reappraised their temporal success to down-regulate positive emotion. Other studies showed that those in the Eastern culture, including Japan, are likely to perceive negative aspects of a positive event or emotion [44, 45]. Overall, individuals in the East are likely to use reappraisal after experiencing both negative and positive events. Therefore, the association between reappraisal and negative aspects, such as negative emotion, found in previous research in the West, may be expected to be weakened in the East. In partial support for this view, we found a weak correlation between reappraisal and psychological distress (see Table 2). In sum, this study suggests that considering overall frequency in the use of reappraisal may contribute little to AEB levels in Asia.

Additionally, methodological differences may underlie the inconsistent findings regarding reappraisal and AEB. One cross-sectional study showed a negative association between reappraisal and frequency of food restriction; in that study, however, no other ERSs were measured and controlled [17]. Controlling for other strategies, the regression analysis in this study revealed a non-significant association between the use of reappraisal and the drive for thinness. These findings may reflect that any associations between reappraisal and symptoms of anorexia in prior studies without controlling other ERSs are spurious. Supporting this view, a prior study has reported no differences in the frequency of using reappraisal between anorexia patients without bulimic symptoms and healthy controls [46]. In addition, the findings in this study suggest that reappraisal functions as a suppressor variable, such that the coefficient between reappraisal and bulimic symptoms is positive in the correlation analysis, whereas the coefficient in the regression analysis becomes negative. Taken together, to clarify relationships between an individual ERS and AEB, it is necessary to control other ERSs simultaneously. Therefore, with taking account of the effects of other ERSs, reappraisal seems to contribute little to levels of anorexic and bulimic symptomatology among women.

### Associations between automatic maladaptive ERSs and AEB

Consistent findings were found across the correlation and regression models regarding the associations of brooding with the drive for thinness and bulimic symptoms. Both analyses revealed that women prone to brooding exhibited more severe levels of the two symptoms with small to medium effect sizes (i.e. β = 0.171–0.301). Particularly, the coefficient on bulimic symptoms in the regression analysis appeared to be over and above those of the other variables. In line with this finding, prior research has consistently reported that brooding is related to elevated AEB [25, 47]. Therefore, regardless of the frequent use of other ERSs, greater use of brooding is linked to increased AEB levels among women.

This may be explained by the view that rumination, particularly brooding, could function as avoidance. Some researchers have noted that rumination functions as an escaping behaviour since it prevents individuals from confronting emotionally laden topics and engaging in active problem solving [48, 49]. In line with this view, one cross-sectional study has reported positive moderate correlations between brooding and avoidant behaviours among female undergraduates [48]. It is also indicated that AEB function as strategies to avoid aversive affective states [50]. Considering these facts, both AEB and brooding appear to function as avoidant behaviours—thus, brooding seems to be more relevant to AEB levels than the other ERSs.

Reflection was found to be moderately and positively correlated with the drive for thinness and bulimic symptoms (*r* = 0.335–0.433). However, the regression analyses revealed that greater reflection was significantly associated with severe bulimic symptoms with a small effect size (i.e. β = 0.127), whereas no significant association was found with the drive for thinness. Contrary to these findings, previous studies reported that reflection was not associated with overall AEB among adults with eating disorders, healthy adults, and undergraduate students [24, 25, 47]. Based on the findings in this study, the previous findings may be explained by the difference in the degree of association between reflection and anorexic and bulimic symptoms. As such, a non-significant association between reflection and drive for thinness may attenuate the degree of the association between reflection and bulimic symptoms. In other words, considering overall AEB rather than each symptom as an outcome variable, the significant association between reflection and bulimic symptoms may be dissolved. Therefore, this study has extended previous research by providing evidence that reflection is associated with one facet of AEB—bulimic symptoms—but not with all facets of AEB.

Cross-sectional studies in Western cultures revealed positive associations between expressive suppression and AEB [18]. Likewise, contrary to our expectation, the regression analyses indicated a positive association of expressive suppression with bulimic symptoms with a small effect size (i.e. β = 0.108), even though expressive suppression was positively correlated with both anorexic and bulimic symptoms. These findings suggest that expressive suppression is a maladaptive strategy against eating behaviours among women in the East. Supporting this view, one study among Japanese individuals revealed that frequent use of expressive suppression exacerbated feelings of interpersonal frictions and psychological distress [51]. Therefore, like in Western cultures, in the East, expressive suppression is a maladaptive strategy, at least in terms of its association with elevated bulimic symptom levels among women.

The finding that expressive suppression was uniquely associated with bulimic symptoms but not with the symptoms of anorexia may be explained by the notion that anorexic and bulimic symptoms have different emotional valences. Qualitative data among patients with anorexia has indicated that restraint, which is one of the symptoms specific to anorexia, is likely to begin at a time when persons, and their lives, feel out of control [52], suggesting that food restriction may relate to sense of control— positive valence. Moreover, severe food restriction is relevant to high self-esteem [53]. On the other hand, bulimic symptoms are likely to have negative valence. As such, individuals are likely to experience binge eating as embarrassing [53] and socially undesirable [54]. Furthermore, studies across cultures revealed that individuals are more likely to avoid negative emotions than positive ones [55]. Based on these findings, it seems that expressive suppression is more likely to relate to bulimic symptoms with negative emotional valence than symptoms of anorexia with positive ones.

### Clinical implication

The results of the regression analyses regarding the association between rumination, especially brooding, and AEB may be applied to the intervention to alleviate AEB levels among adult women. Previous studies have reported that greater mindfulness decreases the level of rumination and psychological distress [56], which was also associated with elevated AEB (see Table 3). Therefore, popularisation of training to enhance mindfulness in the community may lead to effective prevention of AEB in adult women. However, if such interventions are called ‘prevention of eating pathology’, some adult women may be hesitant to participate in the interventions due to the stigma against psychopathology. Hence, such interventions should be provided within the community in an appropriate way that increases their likelihood to be accepted by most adult women. Considering these notions, it seems that yoga exercises, which involve meditation like mindfulness training [57], can be an appropriate, accepted intervention. In fact, an online survey conducted in Japan revealed that more than 20% of women (20s–30s) practice yoga exercises regularly [58]. Previous studies have indicated yoga exercise cultivates mindfulness and alleviates depression and anxiety [57, 59, 60]. Considering these data, yoga exercises may play a role in preventing AEB among adult women in the community. Therefore, it is expected that future research would focus on developing a yoga exercise targeting the prevention of AEB, such as yoga exercises including psychoeducation regarding eating pathology.

### Limitations

The findings of this study should not be interpreted without considering the following limitations. The sample in this study was non-clinical, and more than half of the participants were in their 40s and 50s. The degree of the correlation between ERSs and AEB has been reported to differ between clinical and non-clinical samples [18] and the onset of eating disorders often occurs in adolescence [1]. These empirical findings may, therefore, weaken clinical suggestions.

However, regardless of the sample’s characteristic, this study can still provide academic value. Because of prolonged courses in eating disorders [61], it is critical to develop effective preventive interventions as well as treatments for eating disorders. Thus, this study, given its non-clinical sample, can contribute to the development of such preventions. In addition, the sample in this study was recruited based on Japan’s population distribution, which may support the applicability of the findings in this study throughout Japan. One may argue that the results in this study may not be valid in other Eastern countries; however, because some adult women in other Eastern countries develop eating disorders [62], the present results may still provide an insight on the associations between ERSs and AEB in the East.

Further limitations are as follow. First, owing to the cross-sectional design, we could not make causal inferences; prospective studies are required to clarify the associations in this study. If the results are replicated in longitudinal designs, the importance of modifying maladaptive ERSs to prevent and treat AEB in women can be used to provide more empirical suggestions. Second, this study relied on self-report measures, which may be vulnerable to inaccurate reporting. For example, even though this study was anonymous, some participants might have reported a lower weight than their actual weight. Third, the study data may be influenced by the unique cultural background of collectivism in East Asia. Prior studies in East Asia have reported differences in scores on several subscales of the EDI [63]. In addition, although we used the drive for thinness and bulimic symptoms as the dependent variables, there are other components of AEB. Further studies should examine the associations of ERSs with these symptoms of eating disorders.

The findings from this study may lead to potential directions for future research. It should not be assumed that the findings of this study apply to men. While recent studies have reported an increase of AEB in men [64], previous studies indicated qualitative differences in AEB between women and men [65]. Therefore, additional studies are required to examine the associations between ERSs and AEB in men.

Furthermore, the regression analyses revealed that socio-demographic variables (i.e. age and BMI) were related to AEB. Although this study did not focus on the associations between other socio-demographic variables (i.e. partner status, having children, and levels of household income) and AEB, previous research has suggested that such variables may contribute to increased AEB [5]. Therefore, in future research, it is necessary to examine the contribution of demographic variables to the levels of AEB.

## Conclusions

The current study yielded two findings. First, after controlling for confounding variables, only brooding was associated with the drive for thinness exhibited by women in their 20s to 50s. Second, compared to the drive for thinness, bulimic symptoms were more likely to be associated with ERSs, with brooding indicating a larger coefficient than other strategies. These results suggest that ERSs are more associated with bulimic than anorexic symptoms, and brooding is a more relevant ERS to AEB levels than other ERSs. Nevertheless, further research is required to clarify the causal relationship between ERSs and AEB.

## Data Availability

The datasets generated and/or analysed during the current study are available from the corresponding author on reasonable request.

## Abbreviations

AEB: Abnormal eating behaviours/attitudes
EAT-26: Eating Attitudes Test-26
ERSs: Emotion regulation strategies
J-EDI-91: Japanese version of the Eating Disorder Inventory
J-RRS: Japanese version of the Rumination Response Scale
J-ERO: Japanese version of the Emotion Regulation Questionnaire
K6-J: Kessler 6 Japanese version
